# Isoalantolactone Induces Cell Cycle Arrest, Apoptosis and Autophagy in Colorectal Cancer Cells

**DOI:** 10.3389/fphar.2022.903599

**Published:** 2022-05-12

**Authors:** Junkui Li, Peili Zhu, Yifei Chen, Shiqing Zhang, Zhu Zhang, Zhang Zhang, Ying Wang, Xiaoli Jiang, Kaili Lin, Wei Wu, Zhixian Mo, Stephen Cho Wing Sze, Ken Kin Lam Yung

**Affiliations:** ^1^ Department of Biology, Hong Kong Baptist University (HKBU), Kowloon Tong, China; ^2^ Golden Meditech Center for NeuroRegeneration Sciences (GMCNS), HKBU, Kowloon Tong, China; ^3^ School of Traditional Chinese Medicine, Southern Medical University, Guangzhou, China; ^4^ School of Pharmacy, Guilin Medical University, Guilin, China; ^5^ JNU-HKUST Joint Laboratory for Neuroscience and Innovative Drug Research, College of Pharmacy, Jinan University, Guangzhou, China; ^6^ School of Public Health, Guangzhou Medical University, Guangzhou, China

**Keywords:** isoalantolactone, colorectal cancer, cell cycle arrest, apoptosis, autophagy, AKT/mTOR signaling

## Abstract

Colorectal cancer (CRC) is an aggressive cancer. Isoalantolactone (IATL) has been reported to exert cytotoxicity against various cancer cells, but not CRC. In this study, we explored the anti-CRC effects and mechanism of action of IATL *in vitro* and *in vivo*. Our results demonstrated that IATL inhibited proliferation by inducing G0/G1 phase cell cycle arrest, apoptosis and autophagy in CRC cells. Repression of autophagy with autophagy inhibitors chloroquine (CQ) and Bafilomycin A1 (Baf-A1) enhanced the anti-CRC effects of IATL, suggesting that IATL induces cytoprotective autophagy in CRC cells. Mechanistic studies revealed that IATL lowered protein levels of phospho-AKT (Ser473), phospho-mTOR (Ser2448), phospho-70S6K (Thr421/Ser424) in CRC cells. Inhibition of AKT and mTOR activities using LY294002 and rapamycin, respectively, potentiated the inductive effects of IATL on autophagy and cell death. *In vivo* studies showed that IATL suppressed HCT116 tumor growth without affecting the body weight of mice. In consistent with the *in vitro* results, IATL lowered protein levels of Bcl-2, Bcl-XL, phospho-AKT (Ser473), phospho-mTOR (Ser2448), and phsopho-70S6K (Thr421/Ser424), whereas upregulated protein levels of cleaved-PARP and LC3B-II in HCT116 tumors. Collectively, our results demonstrated that in addition to inhibiting proliferation, inducing G0/G1-phase cell cycle arrest and apoptosis, IATL initiates cytoprotective autophagy in CRC cells by inhibiting the AKT/mTOR signaling pathway. These findings provide an experimental basis for the evaluation of IATL as a novel medication for CRC treatment.

## Introduction

Colorectal cancer (CRC) is the third most commonly diagnosed form of cancer worldwide (Sung et al., 2021). In 2020, there were over 1.9 million new CRC cases and 0.935 million CRC deaths globally. The high incidence of CRC causes a growing global public health challenge. Treatment options such as surgery, radiotherapy, immunotherapy, targeted therapy and palliative chemotherapy have effectively inhibited CRC progression (Xi and Xu, 2021). Nonetheless, the overall 5-year survival rate of CRC remains unacceptably low due to the limitations of these therapeutic modalities, such as low response rates, severe side effects and the emergence of drug resistance (Xie et al., 2020). Therefore, novel, effective and safe therapeutic options for treating CRC are urgently needed.

Natural products are invaluable sources for the development of new drugs (Newman and Cragg, 2020). They are often used either as drug leads or as actual drugs. Isoalantolactone (IATL) is a sesquiterpene lactone naturally occurring in the roots of *Inula helenium* L. IATL has been reported to exert cytotoxic effects against a variety of cancer cells, including pancreatic cancer (Zhang et al., 2021), esophageal cancer (Lu et al., 2018; Wen et al., 2018), hepatocellular carcinoma (Kim et al., 2021), prostate cancer (Rasul et al., 2013; Chen et al., 2018; Huang et al., 2021) and glioblastoma (Xing et al., 2019). The anticancer effects of IATL mainly involve the induction of apoptosis, cell cycle arrest and autophagy (Weng et al., 2016; Chen et al., 2018; Lu et al., 2018). However, the anti-CRC effects and mechanisms of action of IATL remain elusive.

Autophagy is a lysosome-dependent catabolic pathway by which damaged or senescent organelles are removed. It plays an important role in the regulation of cancer progression and determines the response of tumor cells to chemotherapeutic agents (Yun and Lee, 2018). The role of autophagy in cancer treatment is controversial (Levy et al., 2017). In some situations, it has a cytoprotective effect, while in others, it has a cytotoxic effect (Ye et al., 2021). The cytotoxic effect of autophagy leads to type II programmed cell death, called autophagic cell death. In contrast, cytoprotective autophagy prevents cells from death. Blocking cytoprotective autophagy enhances the anticancer efficacy of chemotherapies. Targeting cytoprotective autophagy has been proposed as a smart strategy for cancer treatment (Liu et al., 2020). Therefore, elucidating the exact role of autophagy in determining the responses of CRC cells to anti-CRC chemotherapeutics will hasten the development of novel therapeutic strategies for CRC.

In this study, we found that IATL suppressed CRC cell proliferation and tumor growth *in vitro* and *in vivo*. In addition to inducing cell cycle arrest and apoptosis, IATL initiated autophagosome formation and complete autophagic flux in CRC cells via suppressing the AKT/mTOR signaling pathway. Inhibiting AKT/mTOR signaling using LY294002 enhanced the anti-CRC effects of IATL, indicating that suppressing AKT/mTOR signaling also contributes to IATL-mediated cell death. The autophagy induced by IATL displayed a cytoprotective role in CRC cells. Blocking autophagy with autophagy inhibitors augmented the cytotoxicity of IATL against CRC cells. Thus, the strategy of combing IATL with an autophagy inhibitor might represent a potential strategy for treating CRC.

## Materials and Methods

### Reagents and Chemicals

**FIGURE 1:**
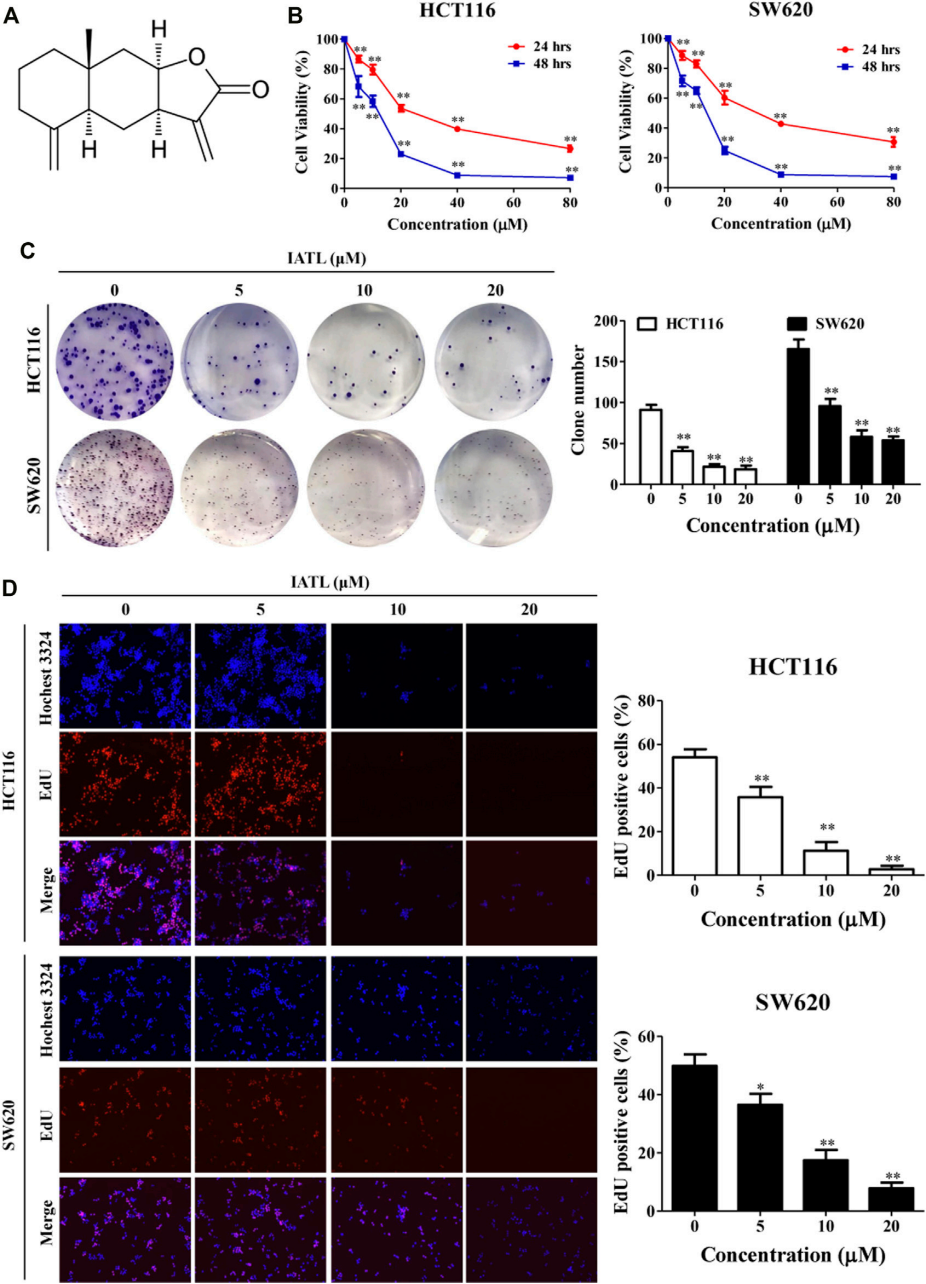
IATL inhibits the proliferation of CRC cells. **(A)** Chemical structure of IATL. **(B)** HCT116 and SW620 cells were treated with various concentrations of IATL for 24 or 48 h. Cell viability was measured using MTT assays. **(C)** IATL inhibited the colony formation ability of HCT116 and SW620 cells. **(D)** Effect of IATL on the proliferation of HCT116 and SW620 cells was examined using the EdU staining assays. The nuclei were stained using Hoechst33342. The percentage of EdU-positive cells are presented in the right panels. Data are shown as mean ± SD of three independent experiments, **p < 0.01 *vs.* vehicle control.

Primary antibodies against p21, CDK2, Cyclin D1, CDK6, Mcl-1, Bcl-xL, Bcl-2, Bax, Poly (ADP-Ribose) Polymerase (PARP), phospho-AKT (Ser473), AKT, phospho-mTOR (Ser2448), mTOR, phospho-70S6K (Thr421/Ser424), 70S6K and LC3B were purchased from Cell Signaling Technology (Beverly, MA, United States). An antibody against β-actin was purchased from Santa Cruz Biotechnology (Santa Cruz, CA, United States). The secondary antibodies, including goat anti-rabbit IgG-horseradish peroxidase (HRP) and goat anti-mouse IgG-HRP, were obtained from Bio-Rad (United States). Dimethyl sulfoxide (DMSO), 3- (4,5-dimethylthiazol-2-yl)-2,5-diphenyltetrazolium bromide (MTT), 3-methyladenine (3-MA), chloroquine (CQ) LY294002, bafilomycin A1 (Baf-A1) and rapamycin (Rapa) were purchased from Sigma Company (St. Louis, MO, United States). IATL with the purity≥98% as determined by high performance liquid chromatography was bought from Chengdu Must Bio-Technology Co. Ltd. (Chengdu, China). The chemical structure of IATL is shown in Figure 1A.

### Cell Culture

Human CRC cell lines HCT116 and SW620 were purchased from the American Type Culture Collection (ATCC, Manassas, VA, United States). CRC cells were cultured in Dulbecco’s modified Eagle’s medium (DMEM, Gibco, Waltham, MA, United States) containing 10% Fetal Bovine Serum (FBS, Gibco, Waltham, MA, United States) and 1% penicillin/streptomycin (Gibco, Waltham, MA, United States). Cells were grown in an incubator at 37°C in a humidified atmosphere containing 5% CO_2_.

### Cell Viability Assay

Cytotoxic effects of IATL against CRC cells were assessed using MTT assays in accordance with an existing protocol (Zhu et al., 2019). After being plated in 96-well plates (at a density of 5,000 cells per well), cells were treated with IATL (5, 10, 20, 40, and 80 μM) for 24 or 48 h. Then, 10 μl of MTT solution (5 mg/ml) was added to each well. After a 2-h incubation, the solution in each well was removed. The formed formazan crystals in each well were dissolved in 100 μl of DMSO. The optical density (OD) was measured at 570 nm using a microplate spectrophotometer (BD Biosciences, San Jose, CA, United States).

### Colony Formation Assay

Cellular proliferation was determined using colony formation assays (Jiang et al., 2022). Briefly, HCT116 and SW620 cells were plated in 6-well plates at a density of 600 cells per well. After being treated with 5, 10, and 20 μM of IATL for 24 h, the cells were cultured with fresh medium for ∼12 days. Thereafter, the colonies were fixed with 4% formaldehyde (PFA) for 15 min, stained with 0.5% crystal violet for 15 min. The violet-stained colonies were counted and then captured.

### EdU (5-Ethynyl-2′-deoxyuridine) Incorporation Assay

Effects of IATL on the proliferative capacities of CRC cells were assessed using a Cell-Light^TM^ EdU Apollo^®^567 EdU *In Vitro* Imaging Kit (Ribobio, Guangzhou, China) according to the manufacturer’s instructions (Jiang et al., 2022). Briefly, CRC cells were seeded in 4-well plates at a density of 4 × 10^4^ cells/well, and then treated with IATL (0, 5, 10, and 20 μM) for 24 h. Subsequently, CRC cells were incubated with 10 μM EdU for 2 h. After fixed with 4% PFA for 30 min, the cells were incubated with 2 mg/ml glycine and subsequently permeabilized with 0.5% Triton X-100 for 10 min. The cells were then incubated with click reaction buffer for 30 min at room temperature under protection from light. Next, the cell nuclei were stained by Hoechst33342 for 30 min at room temperature. The fluorescence images were captured under an inverted fluorescent microscope (Nikon, Japan). The percentage of EdU-positive stained cells was calculated from 5 microscopic fields that were projected at random.

### Cell Cycle Analysis

Both HCT116 and SW620 cells were seeded in 6-well plates at a density of 2 × 10^5^ cells/well. After treatment with various concentration of IATL (5, 10, and 20 μM) for 24 h, the cells were harvested, washed with phosphate-buffered saline (PBS) and gently fixed with 70% ice-cold ethanol at −20°C overnight. Cells were then resuspended in staining buffer (0.5 ml) containing propidium iodide (PI) (25 µl) and RNase A (10 µl), and incubated at room temperature in the dark for 30 min. Afterward, cell cycle distribution was analyzed using a flow cytometer (BD Biosciences, United States) with 10,000 events recorded (Zhu et al., 2019).

### Apoptosis Assay

The effect of IATL on apoptosis was examined using an Annexin V/PI Apoptosis detection kit. Briefly, CRC cells (2 × 10^5^ cells per well) were seeded in 6-well plates and treated with various concentrations (5, 10, and 20 μM) of IATL for 24 h. Both adherent and detached cells were collected and washed twice with PBS. The cells were then resuspended in 100 µl of 1 × binding buffer containing 5 µl of FITC Annexin V and 5 µl of PI. After 30 min of incubation at room temperature in the dark, 400 μl of 1 × binding buffer was added to stop the reaction. Flow cytometric analyses of the samples were performed on a FACSCaliburTM system (BD, San Jose, CA, United States) utilizing 10,000 events (Su et al., 2017).

### Western Blot Analysis

Immunoblotting of protein samples prepared from CRC cells or tumor tissues was performed following a previous study (Zhu et al., 2019). The protein samples were separated by 10–12.5% sodium dodecyl sulfate-polyacrylamide gel electrophoresis and electrotransferred to polyvinylidene fluoride membranes (Millipore, Bedford, MA, United States). The membranes were then blocked with 5% (w/v) skimmed milk for 1 h and incubated with corresponding primary antibodies at 4°C overnight. Subsequently, the membranes were washed with Tris-buffered saline buffer with 0.1% Tween-20 (TBST) and incubated with HRP-conjugated secondary antibodies at room temperature for 1 h. After washing with TBST, immunoreactive bands were visualized using enhanced chemiluminescence (ECL) detection kit (Invitrogen, Waltham, MA, United States) following the manufacturer’s instructions. Finally, the grey value of each band was measured using Image J software.

### LC3 Puncta Analysis

After treatment with IATL for 24 h, HCT116 and SW620 cells on coverslips were fixed with 4% PFA for 15 min, permeabilized with 0.1% Triton X-100 in PBS for 10 min, and then blocked with PBS containing 4% bovine serum albumin for 1 h at room temperature. Thereafter, cells were incubated with the LC3B antibody (1:200) overnight at 4°C. Then, cells were incubated with an Alexa Fluor 488-conjugated secondary antibody for 1 h at room temperature in the dark. The cells were then stained with 4’,6-diamidino-2-phenylindole (DAPI) for 5 min. At the end of each step described above, the coverslips were washed with PBS containing 0.01% Triton X-100. Images of the cells were captured using a confocal microscope (Olympus, Japan).

To investigate the effect of IATL on the fusion of autophagosomes and lysosomes in CRC cells, a Premo^TM^ autophagy Tandem Sensor RFP-GFP-LC3B kit (ThermoFisher Scientific, Waltham, MA, United States) was employed. SW620 cells were transfected with the LC3B reagent for 24 h following the manufacturer’s instructions and then treated with IATL or Rapa (positive control) for another 24 h. Subsequently, the cells were fixed with 4% PFA for 15 min and counterstained with DAPI for 5 min. Images of the cells were captured under a confocal microscope (Olympus, Japan).

### *In Vivo* Study

Eight-week-old male BALB/c-nu/nu mice were purchased from the Chinese University of Hong Kong and reared under a 12 h light-dark cycle with free access to food and water. All animal experiments were approved and conducted in accordance with the guidelines of the Committee on the Use of Human and Animal Subjects in Teaching and Research, Hong Kong Baptist University. For the construction of the CRC xenograft mouse model, 3 × 10^6^ HCT116 cells suspended in PBS were subcutaneously injected into the flank of individual BALB/c-nu/nu mice (at 0.1 ml each). Seven days after cell injection, mice were randomly divided into vehicle control group, 10 mg/kg IATL group or 20 mg/kg IATL group, with 6 mice for each group. IATL dissolved in a PBS solution containing 5% PEG400 and 5% Tween80 was intraperitoneally (i.p.) administrated to mice for 15 consecutive days. When tumors became apparent, tumor volumes were measured using a Vernier caliper once every 3 days. Tumor volume was calculated using the formula: length × width^2^/2. Body weight of each mouse was also recorded once every 3 days. At the end of the experiment, mice were euthanized with excessive anesthesia (isoflurane, 5%). Tumors were excised, weighed, and photographed.

### Statistical Analysis

All data were expressed as means ± standard deviation (SD). Data from cell and mouse assays were obtained from three independent experiments and six mice, respectively. Statistical analysis was determined by one-way ANOVA followed by the Dunnett’s multiple comparisons using GraphPad Prism version 5.0 software (United States). *p* < 0.05 was regarded as statistically significant.

## Results

### Isoalantolactone Suppresses the Proliferation of Colorectal Cancer Cells

The cytotoxic effects of IATL against human CRC cell lines were investigated using MTT assays. Results in Figure 1B show that IATL reduced the viabilities of HCT116 and SW620 cells in a dose- and time-dependent manner. To examine whether IATL inhibits the proliferation of CRC cells, both short- and long-term cell proliferation assays were performed using EdU and colony formation assays, respectively. In comparison to the vehicle control group, IATL significantly reduced the number of colonies (Figure 1C) and the percentage of EdU positive cells (Figure 1D). These results indicate that IATL suppresses the proliferation of CRC cells.

### Isoalantolactone Induces Cell Cycle Arrest at the G0/G1 Phase in Colorectal Cancer Cells

**FIGURE 2:**
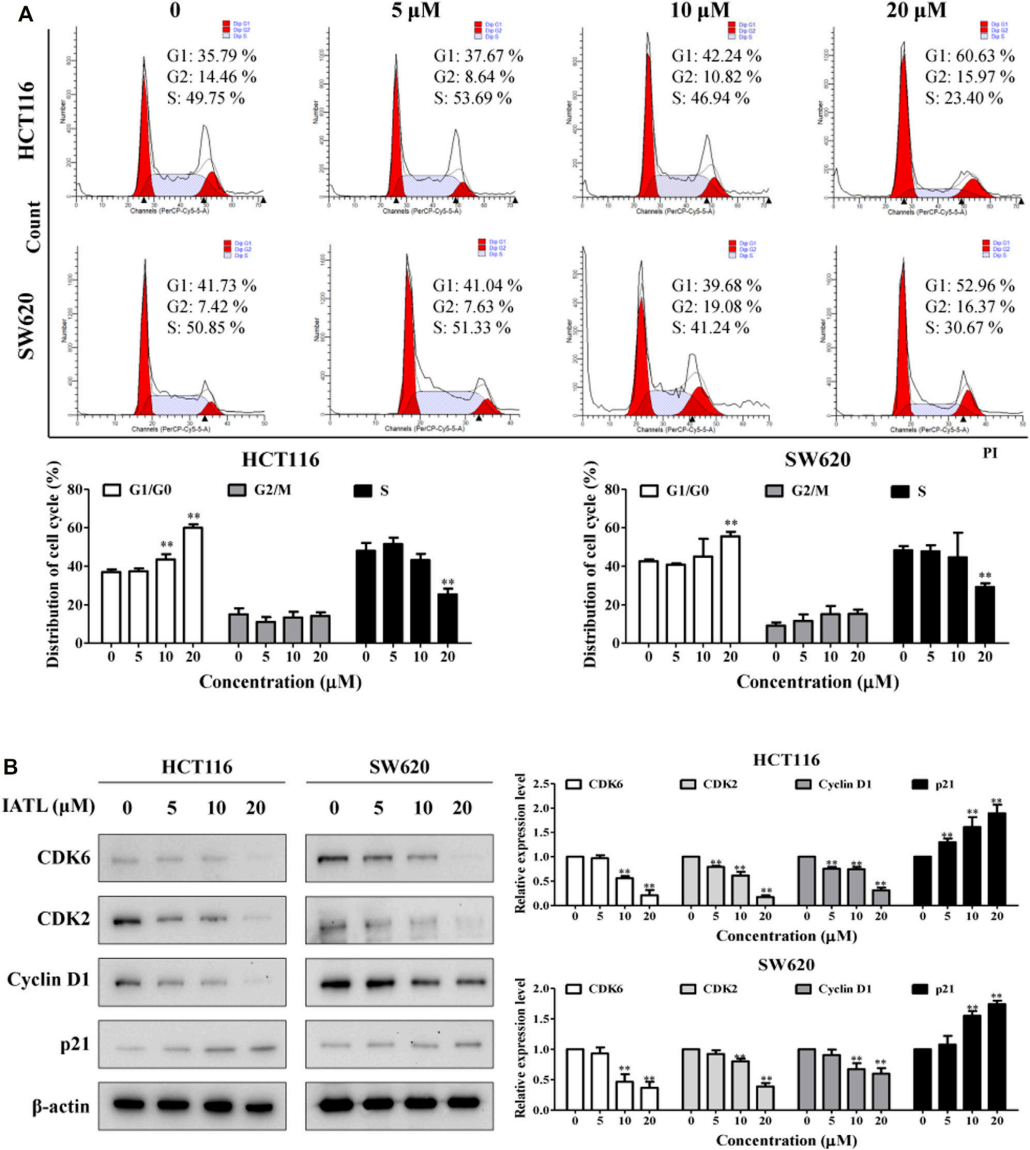
IATL induces G0/G1 phase cell cycle arrest in CRC cells. CRC cells were treated with various concentrations of IATL for 24 h **(A)** IATL induced G0/G1 phase cell cycle arrest in CRC cells. Cell cycle distributions were examined using flow cytometry. **(B)** Effects of IATL on levels of G0/G1 phase cell cycle arrest related proteins. Levels of indicated proteins were determined by immunoblotting. β-actin was included as a loading control. Representative results (left panels) and quantitative results (right panels) are shown. Data are shown as mean ± SD of three independent experiments. *p < 0.05, **p < 0.01, *vs*. vehicle control.

To figure out whether the anti-proliferative effects of IATL are caused by cell cycle arrest, flow cytometric analyses were conducted. Results in Figure 2A show that the number of cells at the G0/G1 phase was markedly increased after being treated with IATL for 24 h, when compared with control group. Correspondingly, the number of cells at S phase was dose-dependently decreased following exposure to IATL. No obvious alterations in G2/M cell population were observed in IATL-treated CRC cells. Cycle-cellular proteins (Cyclin A/D), cyclin-dependent kinases (CDK2/4) and p21 have been reported to play crucial roles in regulating cell cycle progression from the G0/G1 phase to the S phase (Li et al., 2021). Next, we determined whether IATL alters the expression of G0/G1 cell cycle regulator proteins (Kim et al., 2020). Western blotting results showed that IATL lowered the levels of early G1 CDK and cyclin proteins, such as CDK6 and Cyclin D1, and late G1 phase CDK proteins, such as CDK2. Additionally, IATL upregulated the protein level of p21 in both HCT116 and SW620 cells (Figure 2B). These findings indicate that IATL restrains CRC cell cycle at the G0/G1 phase.

### Isoalantolactone Triggers Apoptosis in Colorectal Cancer Cells

**FIGURE 3:**
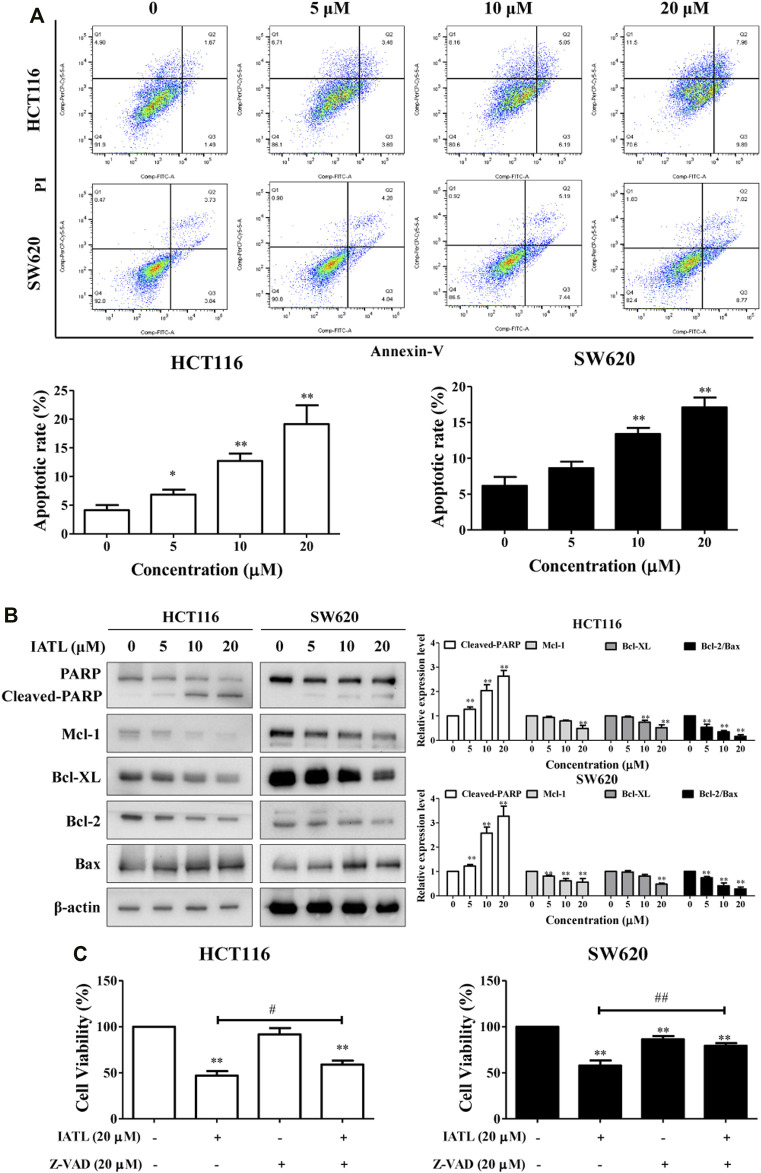
IATL induces apoptosis in CRC cells. HCT116 and SW620 cells were treated with various concentrations of IATL for 24 h. **(A)** Apoptosis was analyzed by flow cytometry following a double staining with Annexin V and PI. **(B)** Protein levels of PARP, Mcl-1, Bcl-XL, Bcl-2, and BAX were examined by Western blotting. Representative (left panels) and quantitative results (right panels) are shown. **(C)** Inhibiting apoptosis with Z-VAD (20 μM) diminished the cytotoxic effects of IATL. Cells were pretreated 20 μM Z-VAD for 1 h, followed by treatment with 20 μM IATL for 24 h. Cell viability was assessed using MTT assays. Data in bar charts are mean ± SD of three independent experiments, *p < 0.05, **p < 0.01 *vs.* vehicle control. #*p* < 0.05, ##*p* < 0.01.

To determine whether apoptosis participates in IATL-mediated inhibition of CRC cell growth, Annexin V/PI double staining was performed. Our results demonstrate that IATL dose-dependently triggered apoptosis in CRC cells (Figure 3A). After being exposed to 5, 10, and 20 μM IATL for 24 h, the apoptotic proportion of HCT116 cells increased from 4.1 ± 0.6% to 11.7 ± 2.7%, 25.2 ± 1.7% and 28.9 ± 1.2%, respectively, and the apoptotic proportion of SW620 cells increased from 3.3 ± 0.4% to 8.8 ± 0.9%, 12.9 ± 1.1%, 20.8 ± 1.3%, respectively. PARP cleavage/activation is a marker of apoptosis. Western blotting results demonstrated that IATL markedly elevated protein levels of cleaved-PARP in CRC cells. The influence of IATL on the expression of apoptosis-related genes, in particular, the Bcl-2 family proteins Mcl-1, Bcl-XL, Bcl-2 and Bax were subsequently explored. We found that IATL lowered antiapoptotic protein Mcl-1, Bcl-XL and Bcl-2 levels, whereas it upregulated pro-apoptotic protein Bax level in CRC cells (Figure 3B). To further confirm the involvement of apoptosis in the anti-CRC effects of IATL, a pan-caspase inhibitor Z-VAD-FMK (Z-VAD) was employed. Pretreatment with Z-VAD for 1 h diminishes the cytotoxic effects of IATL against HCT116 and SW620 cells (Figure 3C). These data suggest that induction of apoptosis contributes to the anti-CRC cells of IATL.

### Isoalantolactone Induces Autophagy in Colorectal Cancer Cells

**FIGURE 4:**
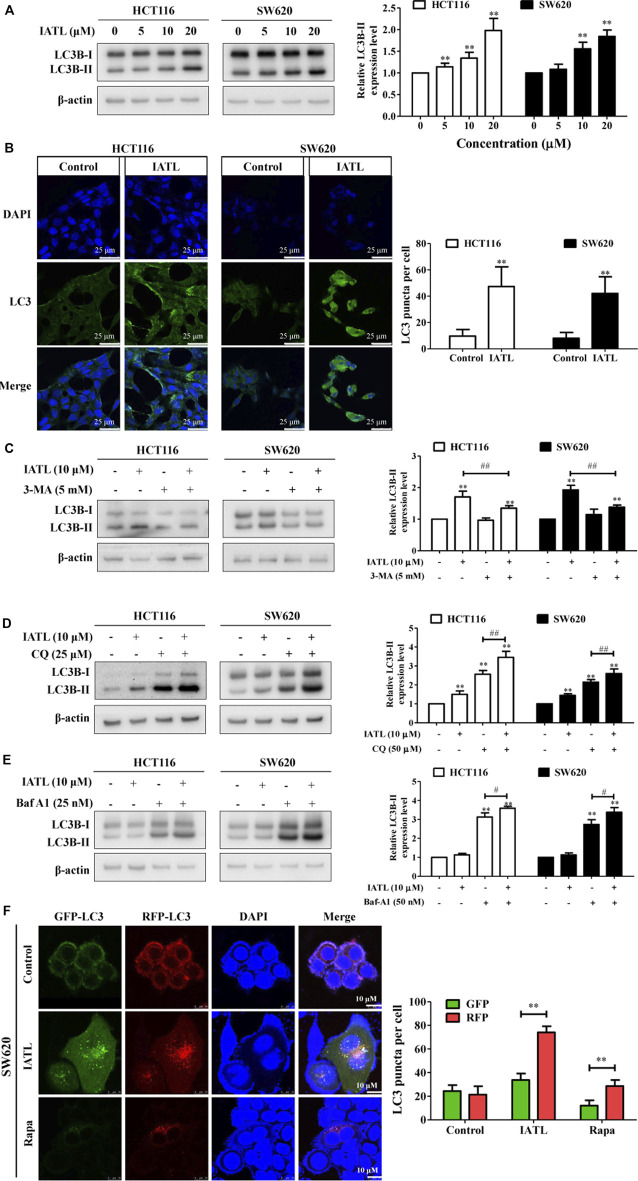
IATL induces autophagy in CRC cells. **(A)** IATL upregulated protein levels of LC3B-II in CRC cells. Representative immunoblotting bands of LC3B and β-actin are presented in left panels and quantitative results for LC3B-II are shown in the right panels. **(B)** IATL increased the numbers of cytoplasmic LC3B puncta in HCT116 and SW620 cells. Cells were treated with 10 μM of IATL for 24 h LC3B puncta were visualized using immunofluorescence analyses. Representative images were photographed under a confocal microscope. Scale bar = 25 μM. Representative images (left panels) and the average numbers of green LC3B dots per cell (right panel) are shown. **(C–E)** Effects of IATL on LC3B-II protein level in the absence or presence of **(C)** 3-methyladenine (3-MA, 5 mM), **(D)** chloroquine (CQ, 25 μM) and **(E)** bafilomycin A1 (Baf-A1, 25 nM) in CRC cells. HCT116 and SW620 cells were treated with indicated drugs for 24 h. Protein levels were examined by immunoblotting. Representative immunoblotting bands are presented in left panels, and quantitative results are shown in right panels. Data in bar charts are presented as mean ± SD of three independent experiments. **(F)** IATL promoted autophagic flux in CRC cells. SW620 cells were transiently transfected with tandem sensor RFP-GFP-LC3B reagent. Rapa (100 nM) was used as a positive control. Representative images were photographed under a confocal microscope. Scale bar = 10 μM. Representative images (left panels) and the average numbers of red and green LC3B dots per cell (right panel) are shown. **p < 0.01 *vs.* vehicle control. ^#^ *p* < 0.05, ^##^ *p* < 0.01.

IATL has been shown to induce autophagy in ovarian carcinoma cells (Weng et al., 2016). Here, we examined whether IATL affects autophagy in CRC cells. LC3B is a specific marker of autophagosome formation in mammalian cells and is widely used as an indicator of autophagy (Mizushima et al., 2010). We found that IATL markedly elevated protein levels of LC3B-II in HCT116 and SW620 cells, indicating that IATL increases the number of autophagosomes in CRC cells (Figure 4A). To confirm that IATL increases autophagosomes in CRC cells, immunofluorescence analyses were performed. In line with the findings demonstrated by Western blotting, we observed an increased accumulation of LC3 puncta in CRC cells treated with 10 μM of IATL, as compared with that in vehicle control-treated cells (Figure 4B). LC3B-II specifically localizes in autophagic structures throughout the autophagic process, from phagophore formation to lysosomal degradation. This implies that both induction of autophagy and inhibition of late-stage autophagy (autophagosome and lysosome fusion) leads to the increased expression of LC3B-II (Parzych and Klionsky, 2014). Next, we investigated whether IATL-mediated autophagosome formation could be inhibited by 3-MA, an autophagy inhibitor that blocks autophagosome formation, in CRC cells. As shown in Figure 4C, IATL-induced increase in LC3B-II protein level was attenuated by 3-MA pretreatment in HCT116 and SW620 cells. To further determine whether IATL promotes autophagosome formation or suppresses autophagosome degradation, CQ and Baf-A1 were used (Zhou et al., 2019). CQ and Baf-A1 are late-stage autophagy inhibitors that block autophagosome-lysosome fusion. Results in Figures 4D,E show that IATL significantly upregulated the protein levels of LC3B-II in CRC cells in the presence of CQ and Baf-A1, when compared with IATL mono treatment. These results indicate that IATL induces complete autophagic flux in CRC cells. This conclusion is supported by Qiu’s report, which demonstrated that if autophagy is induced, co-treatment with CQ will increase the LC3B-II protein level, whereas the protein level of LC3B-II will not be affected in the presence of CQ if autophagy is inhibited at the late stage (Qiu et al., 2014).

To provide more evidence that IATL promotes autophagic flux in CRC cells, we transiently transferred tandem RFP-GFP-LC3B into SW620 cells. Expression of tandem RFP-GFP-LC3B results in both red and green fluorescence. When autophagy is blocked at the degradation stage, colocalization of RFP and GFP punctate fluorescence (merged as yellow) will be observed. However, when autophagy is activated, the red punctate fluorescence will become predominantly visible rather than green punctate fluorescence, because the acidic lysosomes quench GFP fluorescence. Confocal microscopy analyses showed that the number of red fluorescence spots increased predominantly in IATL-treated SW620 cells, compared with that in vehicle control treated cells (Figure 4F). This result validated that IATL initiated autophagy in CRC cells. Similarly, treatment with Rapa (positive control), an autophagy activator, resulted in an increase in the number of red fluorescent spots in SW620 cells (Figure 4F). Overall, these results indicate that IATL enhances autophagosome formation and promotes autophagic flux in CRC cells.

### Inhibiting Autophagy Enhances the Anti-Colorectal Cancer Effects of Isoalantolactone

**FIGURE 5:**
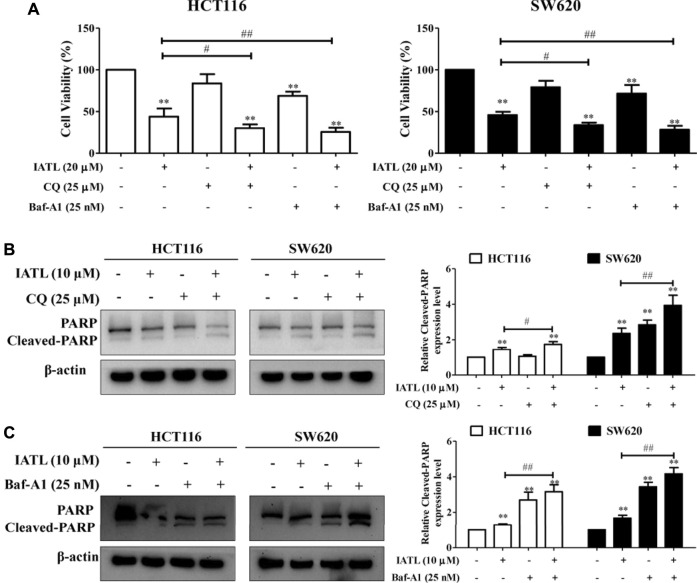
Blocking autophagy enhances the cytotoxic and apoptotic effects of IATL in CRC cells. **(A)** Blocking autophagy with CQ (25 μM) or Baf-A1 (25 nM) enhanced the cytotoxic effects of IATL. Cell viability was assessed using MTT assays. **(B,C)** Blocking autophagy with CQ or Baf-A1 potentiated the apoptotic effects of IATL. Protein level of PARP was detected by immunoblotting. Left and right panels show the representative immunoblotting results and quantitative results, respectively. In **(A)**—**(C)**, CRC cells were treated with IATL in the presence or absence of CQ or Baf-A1 for 24 h. Data in bar charts show mean ± SD of three independent experiments. **p < 0.01 *vs.* vehicle control. ^#^ *p* < 0.05, ^##^ *p* < 0.01.

To elucidate the functional role of autophagy in IATL-mediated cell death, MTT assays were performed. Results in Figure 5A show that blunting autophagy with CQ or Baf-A1 enhanced the sensitivity of CRC cells to IATL treatment, evidenced by decreased viability of CRC cells treated with a combination of IATL and CQ or Baf-A1, compared with IATL mono treatment. In comparison to CRC cells treated with IATL only, CRC cells treated with a combination of IATL and CQ showed greater PARP cleavage, suggesting that suppressing autophagy potentiates the pro-apoptotic effects of IATL (Figure 5B). Similar effects of the co-treatment with Baf-A1 and IATL were detected in HCT116 and SW620 cells (Figure 5C). Taken together, these findings indicate that IATL induces cytoprotective autophagy in CRC cells.

### Inhibition of the AKT/mTOR Signaling Pathway Contributes to Isoalantolactone-Induced Autophagy and Cell Death in Colorectal Cancer Cells

**FIGURE 6:**
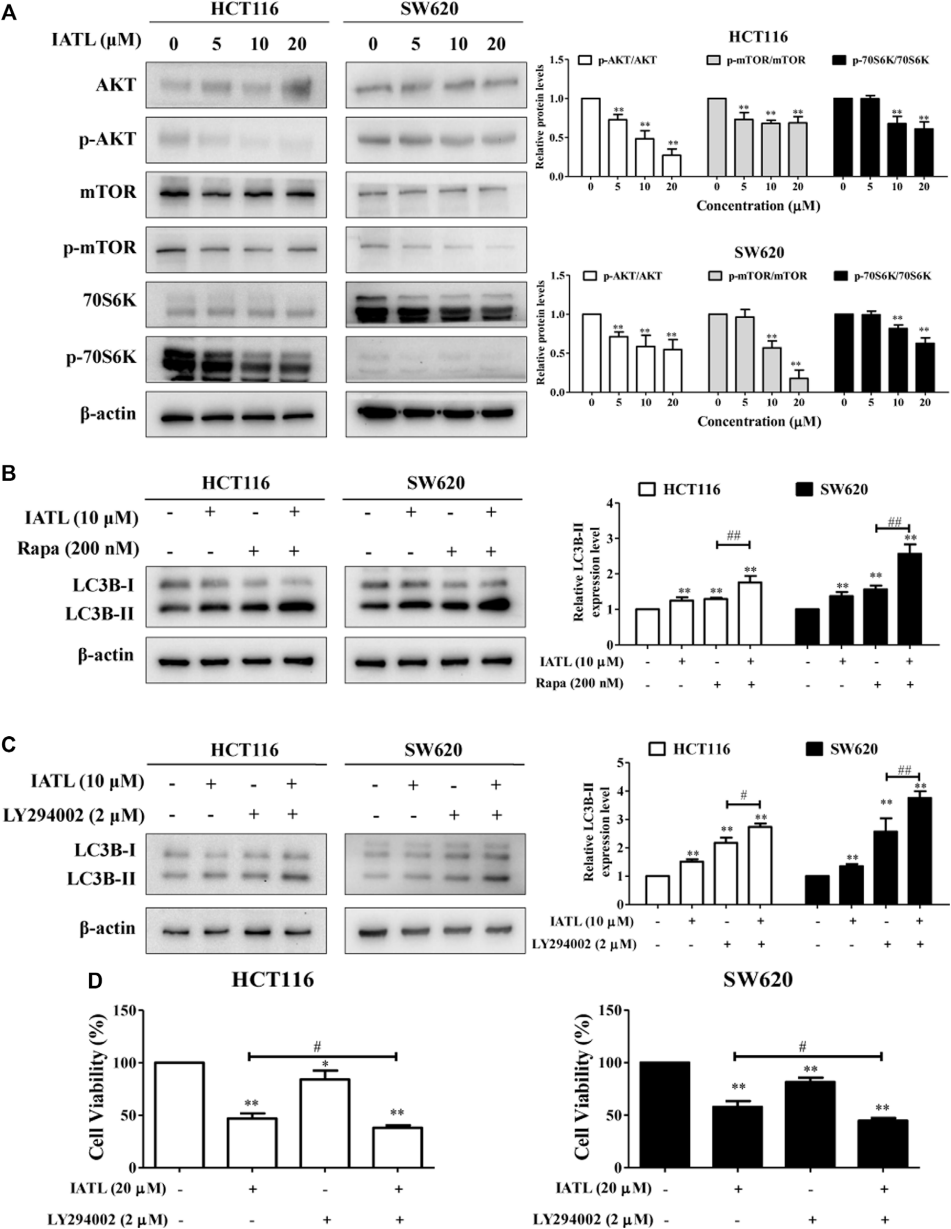
Inhibiting AKT/mTOR signaling contributes to IATL-induced autophagy in CRC cells. HCT116 and SW620 cells were treated with indicated drugs for 24 h **(A)** IATL inhibited AKT/mTOR signaling in CRC cells. Cells were treated with various concentrations of IATL for 24 h. **(B)** Effects of IATL on LC3B-II protein level in the absence or presence of Rapa (100 nM, an mTOR inhibitor) in CRC cells. **(C)** Effects of IATL on LC3B-II protein level in the absence or presence of LY294002 (2 μM, an AKT/mTOR signaling inhibitor) in CRC cells. Protein levels were examined by immunoblotting. β-actin was used as a loading control. Representative immunoblotting bands are presented in left panels, and quantitative results are shown in right panels. **(D)** CRC cells were treated with IATL (20 μM) in the presence or absence of LY294002 (2 μM) for 24 h. Cell viability was assessed using MTT assays. Data in bar charts are mean ± SD of three independent experiments. *p < 0.05, **p < 0.01 *vs.* vehicle control. ^#^ *p* < 0.05, ^##^ *p* < 0.01.

The AKT/mTOR signaling pathway is a canonical negative regulator of autophagy (Dossou and Basu, 2019). We, therefore, investigated whether IATL affects the AKT/mTOR signaling pathway in CRC cells. Western blotting results showed that IATL significantly lowered the protein levels of phospho-AKT (Ser473), phospho-mTOR (Ser2448) and phospho-70S6K (Thr421/Ser424) (a downstream molecule of mTORC1) in CRC cells, when compared with control cells (Figure 6A). To further detect the involvement of mTOR in IATL-induced autophagy in CRC cells, Rapa, an mTOR inhibitor, was employed. In comparison to IATL mono treated cells, protein levels of LC3B-II were significantly increased in HCT116 and SW620 cells that were co-treated with IATL and Rapa (Figure 6B). Furthermore, co-treatment with LY294002, an PI3K/AKT inhibitor, further increased the protein levels of LC3B-II in IATL-treated cells (Figure 6C), suggesting that inhibiting AKT/mTOR signaling is involved in IATL-induced autophagy. In addition to autophagy induction, inhibiting the AKT/mTOR signaling pathway has also been reported to contribute to drug-induced cell cycle arrest and apoptosis, thereby leading to cell death (Zhao et al., 2021). MTT results in Figure 6D show that pretreatment with LY294002 augmented the cytotoxicity of IATL against CRC cells, indicating the involvement of AKT/mTOR signaling in IATL-mediated cell death. Taken together, these data indicate that inhibiting AKT/mTOR signaling contributes to IATL-induced autophagy and cell death in CRC cells.

### Isoalantolactone Suppresses Tumor Growth in HCT116 Cell-Bearing Mice and Suppresses AKT/mTOR Signaling in Tumors

**FIGURE 7:**
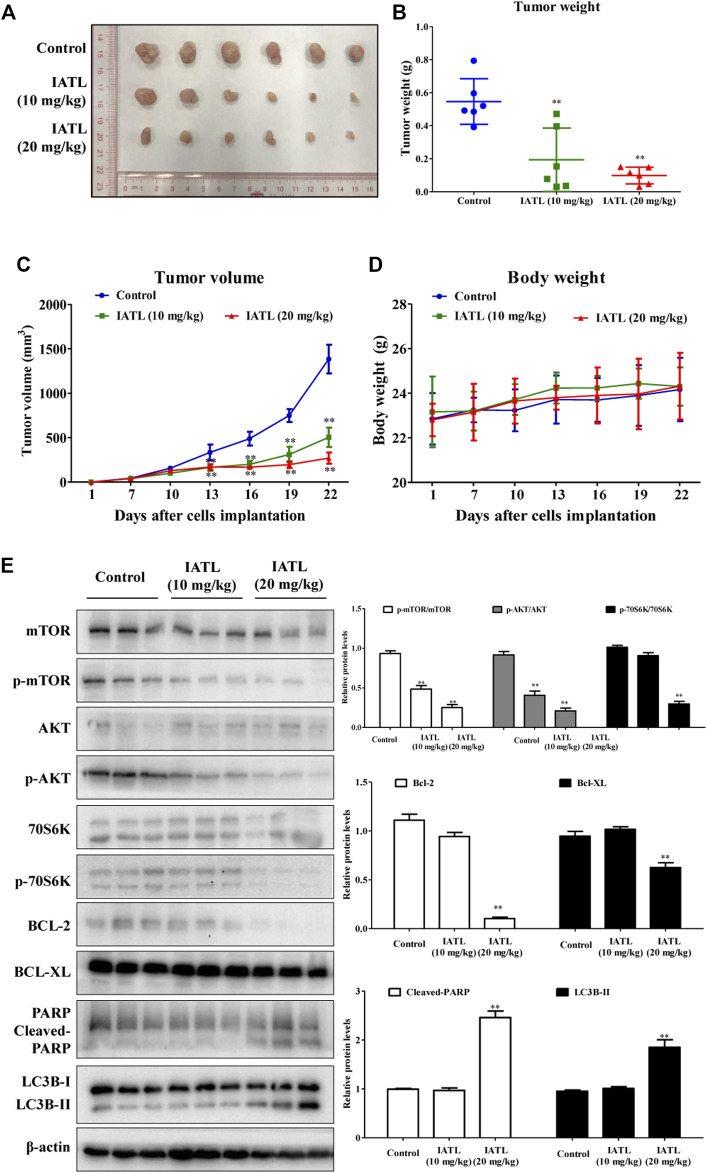
IATL inhibits HCT116 tumor growth in BALB/c-nu/nu mice. Nude mice were i.p. administered with vehicle control (PBS solution containing 5% PEG400 and 5% Tween80), IATL (10 mg/kg), or IATL (20 mg/kg) daily for 15 consecutive days. Photographs of tumors **(A)**, tumor weights **(B)**, tumor volumes **(C)** and mouse body weights **(D)** are shown. Data are presented as mean ± SD of six mice. **(E)** IATL lowered protein levels of phospho-mTOR (Ser2448), phospho-AKT (Ser473), phospho-70S6K, Bcl-2 and Bcl-XL and upregulated protein levels of cleaved-PARP and LC3B-II in HCT116 xenografts. Immunoblotting for xenografts from three individual mice were performed. Representative immunoblotting bands (left panels) and quantitative results are shown (right panels). Data in bar charts are presented as mean ± SD. **p < 0.01 *vs.* vehicle control group.

To determine whether IATL suppresses CRC tumor growth *in vivo*, a HCT116 xenograft mouse model was established. As shown in Figure 7A, IATL dosing for 15 days significantly suppressed tumor growth in HC116 cell-bearing mice. The weights of tumors in 10 mg/kg and 20 mg/kg IATL-treated groups were reduced by 64.5% and 82.1%, respectively, compared with that in the vehicle control-treated group (Figure 7B). After 6 days of treatment, the average volume of tumors in 10 mg/kg and 20 mg/kg IATL-treated groups were markedly smaller than that in the control group (Figure 7C). By day 21, the average tumor volume in 10 mg/kg and 20 mg/kg IATL-treated groups were 36.3% and 18.5% of that of control group. There was no significant difference in body weight among groups (Figure 7D). No animals died during the experimental period. No abnormalities were found either in clinical signs (such as changes in skin, mucous membranes, the occurrence of secretions and excretions and autonomic activity) or necropsy for pivotal organs (heart, liver, spleen, lung and kidneys). Western blotting results showed that IATL significantly lowered the protein levels of Bcl-2 and Bcl-XL, and upregulated protein levels of cleaved-PARP and LC3B-II in HCT116 tumors. In line with the *in vitro* results, the *in vivo* results showed that IATL inhibited phosphorylation of AKT (Ser473), mTOR (Ser2448) and 70S6K (Thr421/Ser424) in HCT116 xenografts (Figure 7E). Overall, these results indicate that IATL inhibits CRC growth *in vivo* and AKT/mTOR signaling in HCT116 tumors.

## Discussion

The general inefficacy in CRC treatment provokes increased interest and motivated studies on novel anti-CRC agents. Natural products have attracted global attention in life science field for its role as a traditional medical intervention and a complementary/alternative form of medicine (Zhang et al., 2019). They offer a wide and promising array of natural resources to facilitate drug discovery and development. *Inula helenium* L is a Chinese herbal medicine that has been traditionally used to treat intestinal tuberculosis. It has also been reported to possess anticancer properties (Zhang et al., 2018). Among the bioactive ingredients isolated from *Inula helenium* L, IATL has been demonstrated to possess considerable biological activities, including anticancer properties (Chun et al., 2018; Yan et al., 2020). Studies have also found that IATL has no overt toxicity on mice (Lu et al., 2018; Huang et al., 2021), suggesting that IATL is safe for consumption. Despite its potential cytotoxicity against a variety of cancer cells, no study on the anti-CRC effects of IATL has been conducted.

Effective anticancer treatments often result in the induction of large amounts of tumor cell death, which is important to lead to tumor eradication. Cell death is primarily divided into programmed cell death and non-programmed cell death. In programmed cell death, apoptosis is the most extensively studied cancer cell death modality. It has been widely reported to prevent the survival of cancer cells (Pistritto et al., 2016). In the present study, we provide the first evidence to show that IATL exerts anti-CRC effects by inducing cell cycle arrest and apoptosis. These results together with previous reports suggest that IATL possesses remarkable anticancer properties that can target a wide range of cancer cells. In addition to inducing cell cycle arrest and apoptosis, we find that IATL induces autophagy in CRC cells. In addition to type I programmed cell death (apoptotic cell death), autophagic cell death is known as a type II programmed cell death. Unlike apoptosis, autophagy appears to have dual roles in cancer treatment. Excessive autophagy has been implicated in autophagic cell death. Nevertheless, autophagy has also been shown to facilitate the survival of tumor cells under stressful conditions induced by cancer treatment (Kocaturk et al., 2019). The cytoprotective effect of autophagy promotes the development of drug resistance (Li et al., 2017). Here, we show that blocking autophagy using CQ or Baf-A1 further reduced the viability of IATL-treated cells, suggesting that IATL initiates cytoprotective autophagy in CRC cells. Therefore, our results suggest that a combination of IATL and an autophagy inhibitor may represent an effective modality for CRC treatment.

mTOR is a protein kinase that has been shown to play a critical role in a diversity of biological processes, such as cell proliferation, autophagy and immunity. The activation of mTOR has been found to promote tumor growth and metastasis. Therefore, mTOR inhibitors are widely explored for cancer therapy (Hua et al., 2019). mTOR exists in two distinct complexes: mTORC1 and mTORC2. mTORC1 is unveiled as the most common upstream autophagy suppression regulator due to its critical role in sensing and integrating growth factor signaling, nutrients, redox and energy levels in cells (Dossou and Basu, 2019). The activity of mTORC1 can be monitored by the phosphorylation of its downstream substrates, including the eukaryotic translation initiation factor 4E-binding proteins (4E-BPs), 70S6K and so others (Roux and Topisirovic, 2018). In the present study, we showed that IATL inhibits mTORC1 activity in CRC cells, as indicated by the lowered protein levels of p-mTOR and p-70S6K after a 24-h treatment with IATL. PI3K/AKT is an upstream control point of mTOR signaling. In line with the effects on mTOR activity, IATL induced the dephosphorylation of AKT in CRC cells. Furthermore, we demonstrated that inhibiting AKT and mTOR activities using LY294002 and Rapa, respectively, potentiates IATL-induced autophagy. Inhibiting AKT/mTOR signaling by LY294002 was also found to further reduce the viability of IATL-treated CRC cells, suggesting that inhibition of AKT/mTOR signaling is in involved in IATL-mediated cell death. These findings suggest that inhibiting AKT/mTOR signaling contributes to IATL-induced autophagy and cell death in CRC cells.

In conclusion, IATL exerts anti-CRC effects in cell and mouse models. Specifically, IATL inhibits the proliferation of CRC cells by inducing cell cycle arrest, apoptosis and autophagy. Suppression of AKT/mTOR signaling is found to contribute to IATL-mediated autophagy induction and CRC cell death. Notably, autophagy initiated by IATL promotes the survival of CRC cells. These findings highlight the potential of IATL as a modern phytotherapeutic agent for managing CRC and provide pharmacological justifications for the use of IATL in CRC treatment.

## Data Availability

The original contributions presented in the study are included in the article/Supplementary Material, further inquiries can be directed to the corresponding authors.
